# Portable electrochemical impedance biosensing with DRT-enabled machine learning for detecting *E. coli O157:H7* in poultry meat

**DOI:** 10.3389/frai.2026.1741144

**Published:** 2026-03-26

**Authors:** Yang Tian, Ziyu Liu, Chaitanya Pallerla, Siavash Mahmoudi, Ramesh Bahadur Bist, Yiting Xiao, Terry Howell, Jeyamkondan Subbiah, Dongyi Wang

**Affiliations:** 1 Department of Biological and Agriculture Engineering, University of Arkansas, Fayetteville, AR, United States; 2 Department of Animal Science, University of Arkansas, Fayetteville, AR, United States; 3 Department of Food Science, University of Arkansas, Fayetteville, AR, United States; 4 Department of Biological and Agricultural Engineering, North Carolina State University, Raleigh, NC, United States

**Keywords:** distribution of relaxation time, electrochemical biosensor, electrochemical impedance spectroscopy, *Escherichia coli O157:H7*, field-deployable sensor, machine learning, partial least squares, portable detection

## Abstract

Ensuring food safety requires rapid and accurate detection of pathogens such as *Escherichia coli O157:H7*. Here, we report a portable electrochemical immunosensor coupled with machine learning (ML) that enables quantitative prediction even when the impedance response is not strictly linear with concentration. The sensor employs protein A-mediated oriented antibody immobilization on a gold electrode and measures target binding using electrochemical impedance spectroscopy (EIS) and cyclic voltammetry. To move beyond single-parameter equivalent-circuit fitting, we apply distribution of relaxation times (DRT) deconvolution to resolve the impedance spectrum into mechanistic contributions associated with charge-transfer kinetics, double-layer charging, and transport-limited (diffusion/Warburg-type) processes, and use these DRT-derived features for concentration inference. Multiple ML models (partial least squares (PLS), Random Forest, histogram-based gradient boosting, support vector regression, ridge regression, and Gaussian process regression) were evaluated using leave-one-concentration-out cross-validation and independent hold-out testing, demonstrating accurate prediction on unseen concentration levels. Validation in poultry meat samples artificially inoculated with *E. coli O157:H7* confirmed applicability in a food-relevant matrix, along with high selectivity against non-target bacteria and stable performance during storage. This work is novel in combining DRT-based mechanistic feature extraction with ML-based inference to deliver a field-deployable immunosensing platform for robust pathogen quantification in complex food samples.

## 1 Introduction

Foodborne pathogens remain a critical global challenge, causing an estimated 600 million illnesses and 420,000 deaths annually (WHO, 2010). Among Shiga toxin-producing *Escherichia coli* (STEC), *O157:H7* is associated with particularly severe outcomes, including hemorrhagic colitis and potential progression to hemolytic uremic syndrome (HUS) (Tserenpuntsag et al., 2005), and its low infectious dose and potential to trigger life-threatening complications (Tian et al., 2025a). Beyond public health, outbreaks impose substantial economic burdens on the food industry, with recalls, liability, and productivity losses collectively amounting to billions of dollars each year (USDA, 2010; Hussain and Dawson, 2013; Ijabadeniyi and Olagunju, 2023). Conventional microbiological assays, though reliable, are inherently slow and infrastructure-dependent, leaving processors and regulators without actionable information during critical windows of food production and distribution (Biswas et al., 2024). Molecular diagnostics such as polymerase chain reaction (PCR) (Kabiraz et al., 2023; Guo et al., 2024) provide improved specificity and turnaround time, yet still require skilled operators, thermal cycling instrumentation, and centralized laboratory facilities, limiting their applicability for on-site testing.

To address these gaps, a wide array of biosensing strategies have been explored, including optical (Tian et al., 2025b; Xu et al., 2013), fluorescence (Kakkar et al., 2023), and surface plasmon resonance platforms (Yashini et al., 2024). While these approaches offer high sensitivity and multiplexing capabilities, they often depend on bulky optics, fragile components, or stringent sample preparation steps that restrict portability and increase costs (Tian et al., 2025a). In contrast, electrochemical biosensors have attracted significant attention as practical alternatives due to their low power consumption, scalability, miniaturization potential, and compatibility with complex food matrices (Flores-Ramírez et al., 2024; Ávila Oliveira et al., 2024; Saldaña-Ahuactzi et al., 2025). Numerous immunosensors for *E. coli O157:H7* have been reported, most commonly quantifying target binding by tracking a single impedance metric (for example, Δ*R*_ct_) and fitting the spectrum with an equivalent circuit model, which can work well in controlled buffers but often becomes less reliable in heterogeneous food matrices where multiple processes overlap.

Among electrochemical modalities, electrochemical impedance spectroscopy (EIS) has gained prominence as a label-free technique capable of probing interfacial charge-transfer and mass-transport phenomena at functionalized electrodes with high sensitivity (Zhang et al., 2025; Bancalari et al., 2024). Unlike amperometric or voltametric methods, which rely on redox activity or enzymatic labels, EIS directly captures changes in interfacial impedance associated with biomolecular recognition events, offering superior versatility for pathogen detection. However, conventional EIS analysis typically relies on equivalent circuit models (ECMs), which suffer from solution non-uniqueness, subjective model selection, and poor resolution of overlapping processes (Zhu et al., 2024; Wang, 2025). As a result, calibration curves derived from a single fitted parameter can exhibit systematic deviations when the dominant contribution shifts across concentration ranges or when matrix-dependent transport and polarization effects become significant, limiting quantitative accuracy and transferability between sensors. Simplified kinetic or empirical models offer alternatives but often obscure diagnostically relevant features, thereby limiting accuracy and interpretability.

The Distribution of Relaxation Times (DRT) framework has emerged as a model-independent approach to address these limitations by mathematically deconvoluting impedance spectra into discrete relaxation processes (Vivier and Orazem, 2022). By attributing distinct timescales to charge-transfer resistance, double-layer capacitance, and diffusion events without presupposing circuit topology, DRT enhances both sensitivity and interpretability, making it particularly attractive for immunosensors where binding, interfacial polarization, and transport limitations can coexist and evolve with surface coverage. Recent studies have applied DRT to living systems such as eukaryotic cells and bacteria, to separate membrane and medium contributions, enhance peak resolution, and enable earlier detection of growth-phase transitions than fixed-frequency or lumped-parameter fits (Lu et al., 2024; Lee et al., 2025; Shi and Kolb, 2020; Jiang and Shrotriya, 2025; Hong et al., 2024). However, practical limitations persist, including electrode-polarization artifacts, finite bandwidth and regularization sensitivity, spectral overlap in complex matrices, and device-to-device variability that degrades portability and quantitative accuracy. Importantly, even when a monotonic DRT feature is available, the feature-to-concentration relationship is often nonlinear across a wide dynamic range, so a single linear calibration may not provide robust prediction on unseen concentration levels. Integrating machine learning with DRT addresses these gaps by fusing full-spectrum and DRT-derived features (peak position/width/area and continuum signatures), learning nonlinear mappings to concentration or class labels, performing calibration transfer and domain adaptation across sensors and sample types, and providing uncertainty quantification for decision support. Compared to prior EIS immunosensors that primarily rely on circuit fitting or a single impedance descriptor, this DRT plus ML strategy explicitly separates mechanistic contributions and uses them as predictive features, enabling robust quantification in the presence of overlapping processes and nonlinear responses.

In this case, we developed a portable biosensing platform that acquires impedance data using a precision potentiostat (AD5941) integrated with a wireless microcontroller (ESP32-S3). The device transmits impedance spectra to a smartphone application for real-time display and storage, after which the data are transferred to a laptop for DRT analysis and machine learning (ML) quantification. We further incorporated protein A mediated oriented antibody immobilization on a gold electrode to improve effective antigen binding at the interface and to support consistent impedance signatures across measurements. Among the ML approaches evaluated, we selected a compact model (PLS, *n* = 3) to balance predictive performance and interpretability, enabling accurate quantification of *E. coli O157:H7* concentrations from the processed impedance features. Overall, the novelty of this work lies in combining DRT-based mechanistic feature extraction with ML-based inference to overcome nonlinearity and process overlap that can limit calibration-only EIS immunosensors in food-relevant matrices.

In this work, we validated the platform using poultry meat samples inoculated with *E. coli O157:H7*. The sensor achieved a limit of detection of 10 CFU/mL, demonstrated negligible cross-reactivity with non-target bacteria, and delivered results within 20 min. These findings underscore the potential of combining model-free impedance analysis with ML-assisted quantification to provide a robust and field-deployable solution for food safety.

## 2 Materials and methods

### 2.1 Materials and reagents

Affinity-purified goat anti-*E. coli O157:H7* antibodies (5.0 mg) were purchased from Kirkegaard & Perry Laboratories, Inc. (Gaithersburg, MD, USA) and aliquoted in 50% glycerin solution to a final concentration of 0.5 mg/10 μL. Protein A from *Staphylococcus aureus* Cowan strain cell walls (5.0 mg) was obtained from Sigma-Aldrich (St. Louis, MO, USA). *Escherichia coli O157:H7* (ATCC 43895), *Salmonella enterica* (ATCC 10708), *Staphylococcus aureus* (ATCC 25923), and *Listeria innocua* (ATCC 33090) were purchased from the American Type Culture Collection (ATCC) (Rockville, MD, USA). Phosphate-buffered saline (PBS, pH 7.4), bovine serum albumin (BSA) and all other chemicals were analytical grade (Sigma-Aldrich, USA). Field chicken meat samples were collected from a local market, stored at 4 °C and used within 24 h.

### 2.2 Electrode functionalization via protein a and antibody immobilization

Screen-printed Au electrodes (DRP-C220AT-U75) were purchased from Metrohm DropSens. For cleaning, the Au sensor was soaked in 1 M NaOH and left for 10 min. Afterward, it was rinsed thoroughly with distilled water. Next, 1 M HCl was used for 5 min, followed by another flush rinse with distilled water and an ethanol wash for 3 times, respectively. The final cleaning step involved flushing electrode with PBS to ensure it was ready for further experiments.

After three PBS washes, electrodes were incubated in certain concentration protein A in PBS for 1 h at 25 °C to orient the Fc region of subsequently applied antibodies. Electrodes were rinsed and then incubated with 50 μg/mL anti-*E. coli O157* antibody in PBS for 2 h at 4 °C. Unreacted sites were blocked with 1% BSA for 1 h, followed by a final PBS rinse prior to measurement.

### 2.3 Poultry sample preparation

Twenty-five grams of chicken meat were homogenized with 100 mL sterile PBS using a stomacher (90 s). Aliquots were spiked with *E. coli O157* to yield concentrations of 10^1^–10^6^ CFU/mL. Then, the samples were centrifuged at 2000x*g* for 10 min and supernatants were collected, diluted in PBS and used directly for impedance assays. Identical preparations of non-target strains were used to assess specificity. Identical preparations of *S. enterica*, *L. monocytogenes*, and *S. aureus* were used as negative controls.

### 2.4 Electrochemical test

To investigate the effect of protein A surface coverage on interfacial transport, Au disk electrodes (4 mm diameter; *A* = 0.126 cm^2^) were first modified with protein A at different concentrations. Electrochemical measurements were performed in a three-electrode configuration with the modified Au as the working electrode, an Ag/AgCl reference electrode, and a Pt counter electrode, using an electrolyte containing 0.1 M KCl and 10 mM K_3_[Fe(CN)_6_]/K_4_[Fe(CN)_6_]. Cyclic voltammetry (CV) was recorded at scan rates of 10, 20, 50, 100, and 200 mV s^−1^. Peak currents (Ip) were extracted after baseline correction and plotted against *v*^1/2^. The apparent diffusion coefficient (*D*_app_) of the ferri/ferrocyanide redox was calculated from the slope (S) of the *i_p_* versus *v*^1/2^ relationship using the Randles–Sevcik equation (298 K):


$$i_{p}=(2.69\times 10^{5})n^{3/2}A{D_{\mathrm{app}}}^{1/2}Cv^{1/2}$$

where *n* = 1 for [Fe(CN)_6_]^3−/4−^, *A* is the geometric area of the Au electrode, *C* is the bulk concentration of the redox species (mol cm^−3^), and *v* is the scan rate (V s^−1^). Here, *D_app_* is reported as an effective parameter that captures diffusion and permeability limitations imposed by the protein A layer at the electrode interface (that is, reduced accessibility of the redox probe through the surface layer), rather than a change in the intrinsic bulk diffusivity of the redox couple.

After optimizing and characterizing the protein A layer, the complete biosensor was constructed by immobilizing antibodies onto the protein A modified Au surface. The functionalized IDE electrode was assembled into a fluidic channel, and 200 μL of prepared poultry sample spiked with bacteria was introduced at a low flow rate followed by a 20 min incubation under static conditions to allow binding. The device was then disassembled and the Au electrode was removed and gently rinsed to reduce nonspecific residues. Electrochemical impedance spectroscopy (EIS) was subsequently performed in a clean redox electrolyte (0.1 M KCl plus 10 mM equimolar K3[Fe(CN)6]/K4[Fe(CN)6]) using the three-electrode configuration (Au IDE working, Ag/AgCl reference, Pt counter). Impedance spectra were acquired from 1 Hz to 200 kHz with a 10 mV (rms) sinusoidal perturbation at open-circuit potential. EIS was collected on bare Au, after each functionalization step (protein A and antibody), and after exposure to poultry samples with and without bacteria to quantify changes.

### 2.5 Device assembly and firmware

A custom printed circuit board (60 × 90 × 1.6 mm) integrates an ESP32-S3FN8 microcontroller (Espressif Systems) and an AD5941 analog front end (Analog Devices). The dual-core 240 MHz ESP32-S3 controls waveform generation via SPI, streams data over WiFi 802.11 b/g/n and Bluetooth Low Energy 5.0. The AD5941 provides impedance measurements from 1 Hz to 200 kHz. Power is supplied by a 3.7 V Li-ion cell. Hardware, firmware, and Android app was using previous published work (Bautista et al., 2025).

### 2.6 Impedance measurement and DRT analysis

DRT deconvolution was performed using the open-source Python package pyDRTtools (Maradesa et al., 2023; Saccoccio et al., 2014; Wan et al., 2015). The measured impedance spectra (1 Hz to 200 kHz) were inverted onto a log-spaced relaxation-time axis spanning *τ* = 1/(2π*f*_max_) to *τ* = 1/(2π*f*_min_). The inversion was solved using the regularized DRT solver implemented in pyDRTtools with consistent settings applied across all samples. From the resulting DRT distributions, three dominant relaxation features were identified and assigned to interfacial charge-transfer kinetics (*τ*₁), double-layer charging (*τ*₂), and transport-limited (diffusion/Warburg-type) processes (*τ*₃). Peak magnitudes (and centroid *τ* values, when used) were extracted and plotted versus bacterial concentration to construct calibration relationships.

### 2.7 Selectivity verification by PCR

Selectivity was confirmed by PCR amplification of the rfbE gene using 2 μL of each post-measurement sample as template. Reactions (25 μL) contained 1 × buffer, 2 mM MgCl₂, 0.2 mM dNTPs, 0.4 μM each primer (forward 5′-GGCATAAGTCTGCGTGGGAA-3′; reverse 5′-ATCGCCGTTGTTGCTGTTGA-3′) and 1 U Taq polymerase. Thermal cycling comprised 95 °C for 3 min; 35 cycles of 95 °C for 30 s, 58 °C for 30 s and 72 °C for 45 s, and final extension at 72 °C for 5 min. Amplicons were resolved on 2% agarose gels and visualized under UV light.

### 2.8 Performance evaluation

Detection limits were evaluated for both the electrochemical readout and the machine learning (ML) concentration prediction. For the electrochemical method, the limit of detection (LoD) and limit of quantification (LoQ) were calculated from the calibration relationship between the selected electrochemical feature and log₁₀(CFU/mL) using LoD = 3.3*σ*/S and LoQ = 10*σ*/S, where σ is the standard deviation of blank or near-blank measurements and S is the slope of the linear fit within the low-concentration linear region. Because 0 CFU/mL cannot be represented on a log scale, σ was conservatively estimated from replicate measurements at the lowest tested concentration (10^1^ CFU/mL). Linearity, dynamic range, and repeatability were assessed across 10^1^–10^6^ CFU/mL using replicate measurements at each level. Specificity was assessed by testing non-target strains, where negligible impedance changes were observed relative to target bacteria, and by PCR verification showing no non-target amplicons. Total analysis time, including sample handling, electrode preparation, impedance measurement, and PCR verification.

### 2.9 Machine learning models and validation strategy

We trained and compared ML models to quantify the concentration of *E. coli O157:H7* in poultry samples from impedance data. The response variable was log_10_(CFU·mL^−1^) over 10^0^–10^6^ CFU·mL^−1^. Inputs to each model were either (i) tabular features engineered from EIS-DRT (e.g., peak positions, widths, amplitudes, peak areas, and selected Nyquist/Bode descriptors) or (ii) full-curve representations reduced to latent variables when required (e.g., PLS).

We benchmarked a suite of regression models representing both linear and nonlinear learning paradigms: Partial Least Squares (PLS) with varying latent components (*n* = 3–6), Ridge regression, Support Vector Regression (SVR, radial basis function kernel), Random Forest, Histogram-based Gradient Boosting (HistGB), and Gaussian Process Regression (GPR, RBF kernel). Model selection and validation were performed with grouped 5-fold cross-validation on the training set and a 35% held-out test set; hyperparameters were fixed *a priori*. Data were first split into a 65/35 train/test partition with random_state = 42. Model assessment on the training portion used 5-fold GroupKFold (n_splits = 5). In this analysis, we evaluated a small set of pre-specified hyperparameters (fixed per model) and compared models under identical splits. All preprocessing (*z*-score scaling) was performed inside the CV pipelines. Performance metrics were RMSE on log10(CFU·mL^−1^) for regression and accuracy for classification; we also recorded *R*^2^/MAE where relevant. The final parameters and number are in Supplementary material.

#### 2.9.1 Nested leave-one-concentration-out (LOCO) cross-validation

To mimic real biosensing deployment scenarios where the sensor encounters concentrations not previously trained upon, we applied a nested Leave-One-Concentration-Out (LOCO) CV strategy. In this approach, each concentration level was held out entirely as the test set in the outer loop, while inner-loop cross-validation optimized hyperparameters. This protocol provides a stringent and realistic measure of a model’s capacity to extrapolate across concentration ranges rather than interpolating between known points.

Model accuracy was evaluated using RMSE and MAE in log₁₀(CFU/mL) units and the Median Fold-Error on the native CFU/mL scale. For each test sample, the fold-error was computed as:


$$\text{Fold}-\text{Error}=10^{\left\{|\hat{y}-y|\right\}}=\max(\frac{C_{\text{pred}}}{C_{\text{true}}},\frac{C_{\text{true}}}{C_{\text{pred}}}\;\;)$$

where *y* and *ŷ* denote the true and predicted log₁₀(CFU/mL), and C_true and C_pred are the corresponding concentrations in CFU/mL. The Median Fold-Error is the median of these fold-error values across the evaluation set. The median was used because fold-errors are typically right-skewed and sensitive to outliers. Thus, the median provides a robust estimate of typical multiplicative prediction error and is straightforward to interpret for practical concentration reporting.

#### 2.9.2 Implementation details

All models were implemented in Python 3.10 using the scikit-learn library. Random Forest and HistGB models were configured with default estimators and tuned tree depth; SVR was optimized for cost (C) and kernel width (*γ*); Ridge regression used regularization tuning; and PLS models were tested across latent dimensions (*n* = 3–6). The GPR model with an RBF kernel was included as a benchmark for probabilistic regression, though it showed limited scalability with the dataset size.

Evaluation was conducted on both hold-out test sets (35% split with 48 data sets) and the nested LOCO scheme. This two-pronged validation allowed us to assess both within-range predictive accuracy and generalization to unseen concentration levels, the latter being the most critical criterion for practical biosensor deployment.

## 3 Results and discussion

**Figure 1:**
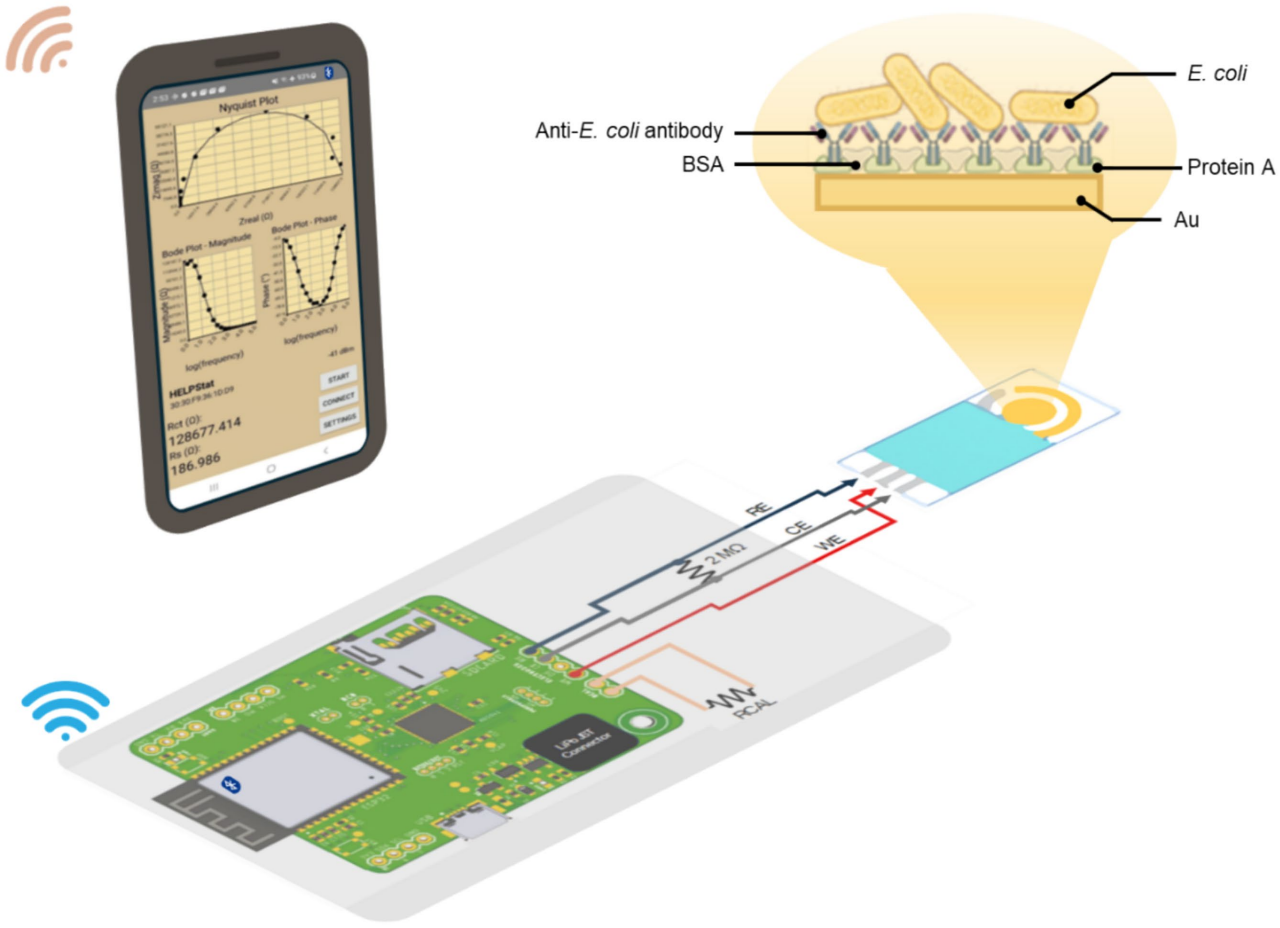
Schematic illustration of the portable biosensing platform. The system integrates a disposable electrochemical sensor strip with a custom-designed circuit board for impedance measurement, powered by a compact microcontroller. Sensor data are transmitted wirelessly to a smartphone, where a dedicated app displays impedance plots in real time, enabling rapid and user-friendly analysis of bacterial concentration in field settings.

In Figure 1, it illustrates the integration of the disposable electrochemical sensor with the custom portable interface and wireless smartphone application. The device acquires impedance spectra using the microcontroller (ESP32)-based potentiostat (AD5941) and transmits the raw data to a smartphone via Bluetooth. The cell phone application displays Nyquist plots in real time and allows the spectra to be stored locally. At the current stage of development, the app provides visualization and data logging only, while advanced processing such as DRT deconvolution and concentration prediction through machine learning is performed offline after transferring the saved spectra to a computer. This workflow enables users to obtain impedance data directly in the field while maintaining compatibility with subsequent model-based analysis.

**Figure 2:**
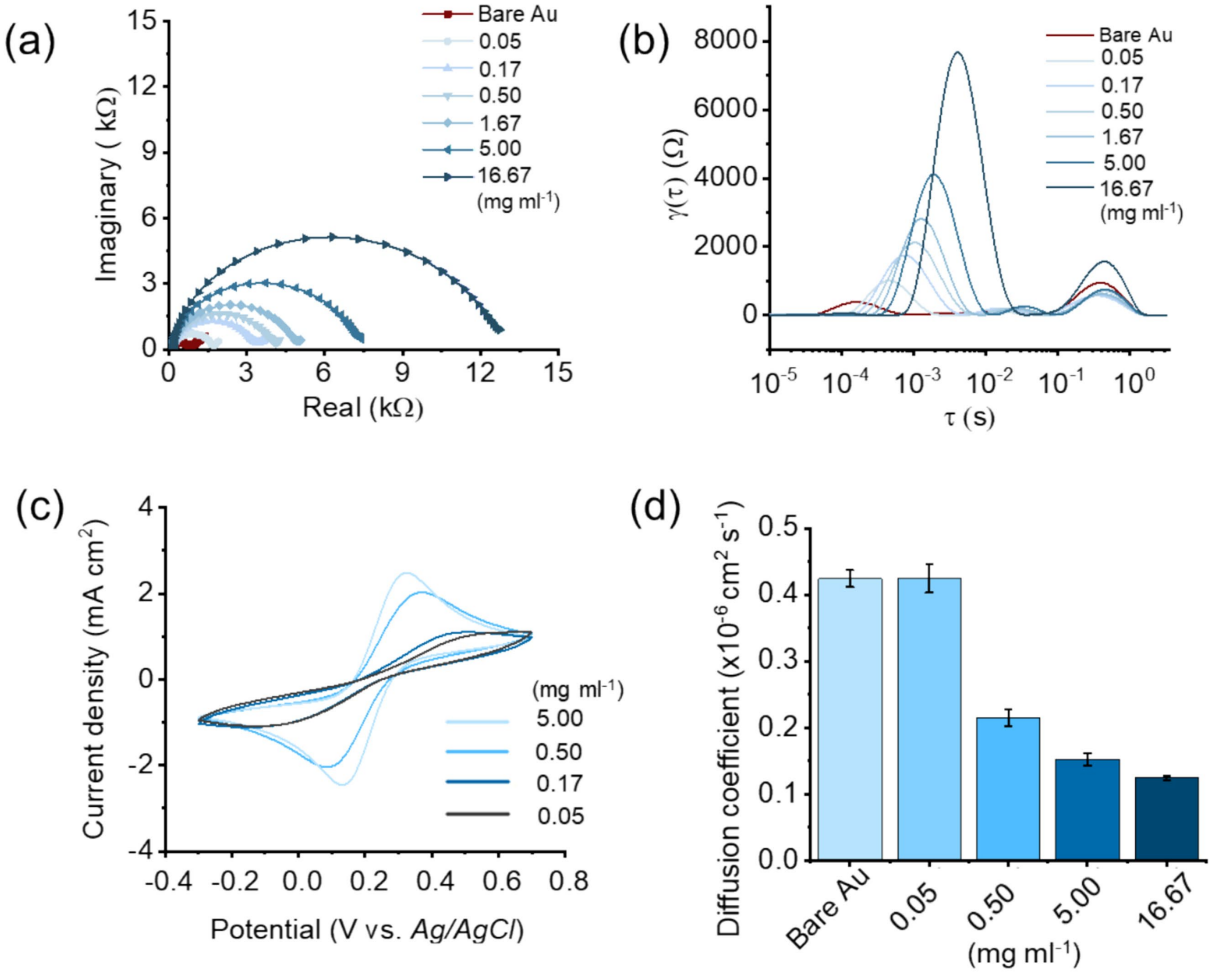
Electrochemical characterization of protein A modification on the gold working electrode. **(a)** Nyquist plots obtained from electrochemical impedance spectroscopy (EIS) at varying coating concentrations of protein A. **(b)** Distribution of relaxation times (DRT) analysis derived from EIS data, illustrating the distinct resistance contributions as a function of protein A loading. **(c)** Cyclic voltammetry (CV) curves at different protein A concentrations, showing changes in electron transfer kinetics upon surface modification. **(d)** Apparent diffusion coefficient (*D*_app_) of K_3_[Fe(CN)_6_]^3−^/K_4_[Fe(CN)_6_]^4−^ through the protein A modified interface estimated from CV.

The electrode modification with protein A was systematically evaluated using a combination of electrochemical impedance spectroscopy (EIS) and cyclic voltammetry (CV). The electrodes coated with protein A were merged in the solution of 0.1 M KCl and 10 m M K_3_[Fe(CN)_6_]^3−^/K_4_[Fe(CN)_6_]^4−^. As shown in the EIS Nyquist plots (Figure 2a), increasing protein A loading on the Au electrode (from the lowest loading at 1000 × dilution to the highest loading at 3×) produces a systematic expansion of the main semicircle along the real axis. In a Randles-type interfacial response, the diameter of this high-to-mid frequency semicircle corresponds to the charge-transfer resistance, Rct, because the semicircle arises from the parallel combination of Rct and the double-layer capacitance (Cdl) in series with the solution resistance (Rs); therefore, a larger semicircle diameter indicates a larger kinetic barrier for electron transfer at the electrode surface. Consistent with this interpretation, the low-frequency real-axis intercept shifts from below 1 kΩ for bare Au and the most dilute protein A conditions to approximately 12 to 13 kΩ at 3×, corresponding to an increase in Rct from sub-kΩ to the low tens-of-kΩ range (Figure 2a). Intermediate loadings show a monotonic trend, with the semicircle diameter increasing to roughly 2 kΩ (300×), 3 kΩ (100×), 4 kΩ (30×), and 7 to 8 kΩ (10×) before reaching the maximum at 3×. This progressive increase in Rct is physically consistent with partial surface blocking and reduced effective electroactive area as the protein layer thickens, which suppresses interfacial electron-transfer pathways and increases the apparent charge-transfer resistance. To better resolve the origins of these impedance changes, the Nyquist data were further analyzed using distribution of relaxation times (DRT) (Figure 2b). Unlike traditional equivalent circuit fitting, DRT provides a model-free approach to deconvolute overlapping processes into distinct peaks corresponding to different relaxation phenomena. For bare electrodes and low protein A coverage, the DRT spectra show sharp, well-defined peaks at short timescales, representing fast electron transfer processes and low interfacial resistance. As protein A loading increases (from low coverage, 0.05–0.5 mg mL^−1^, to the highest loading), the DRT response evolves from a dominant high-frequency charge-transfer feature in the *τ* = 10^−6^–10^−3^ s window into a broader distribution across this same range, and an additional lower-frequency relaxation becomes discernible in the *τ* = 10^−3^–10^−1^ s decade, while at the highest loading (16.7 mg mL^−1^), a further contribution appears at *τ* > 10^−1^ s, consistent with progressively slower interfacial charge-transfer and enhanced dielectric polarization and transport limitations introduced by the thicker protein layer. The broadening and shifting of these peaks clearly demonstrate how protein A immobilization introduces heterogeneous interfacial properties, such as nonuniform coverage, localized insulating domains, and increased double-layer capacitance. These features corroborate the impedance results and provide deeper mechanistic evidence of how protein A loading alters electrochemical behavior.

Unlike equivalent-circuit fitting, DRT decomposes the impedance response into relaxation peaks defined by a characteristic timescale *τ*. In Figure 2b, a dominant short-*τ* feature (*τ* approximately 10^−4^ to 10^−2^ s, centered near 10^−3^ s) is observed and is consistent with charge-transfer-controlled interfacial kinetics. At low protein A loading (0.05 to 0.5 mg mL^−1^) this peak is relatively narrow with a modest amplitude (hundreds to about 1 to 2 kΩ), whereas increasing protein A (1.67 to 5 mg mL^−1^) broadens the peak and increases its amplitude (about 2 to 4 kΩ), indicating more dispersed and slower charge-transfer time constants. At the highest loading (16.7 mg mL^−1^), the peak shifts toward larger *τ* within the same decade and reaches about 7 to 8 kΩ, consistent with strong charge-transfer suppression by a thicker protein layer. A secondary long-*τ* contribution (*τ* approximately 0.2 to 0.6 s) also emerges and grows with protein A concentration, indicative of transport-limited (Warburg-type) behavior. Together, the DRT spectra show a concentration-dependent shift from primarily interfacial polarization effects at low coverage to coupled charge-transfer and mass-transport limitations at high coverage, corroborating the Nyquist trends and demonstrating that excessive protein loading imposes both kinetic and transport penalties.

Cyclic voltammetry (Figure 2c) provided additional insight into the effect of protein A coverage on electrode kinetics. The bare gold electrode exhibited the highest peak currents and most reversible redox (K_3_[Fe(CN)_6_]^3−^/K_4_[Fe(CN)_6_]^4−^) behavior, whereas protein A-coated electrodes showed progressively suppressed peak currents as concentration increased. This suppression is consistent with a reduction in active electrochemical surface area, caused by protein A forming a partial insulating layer. Notably, moderate concentrations maintained redox activity while providing stable surface modification, suggesting a balance between biofunctionalization and conductivity.

The apparent diffusion coefficients (*D*_app_) (Figure 2d) quantitatively highlight the trade-off between biorecognition layer formation and redox probe transport to the electrode. Bare Au and the most dilute protein A condition (0.05 mg mL^−1^) exhibited essentially identical Dapp values, indicating negligible transport penalty at very low coverage. As protein A loading increased, Dapp decreased monotonically, consistent with reduced permeability and steric blocking at the interface: Dapp dropped to 0.21455 × 10^−6^ cm s^−1^ at 0.05 mg mL^−1^ (approximately 49.5% lower than bare Au), to 0.15227 × 10^−6^ cm s^−1^ at 0.5 mg mL^−1^ (approximately 64.1% lower), and to 0.12462 × 10^−6^ cm s^−1^ at 16.7 mg mL^−1^ (approximately 70.6% lower). This progressive reduction indicates that thicker protein A layers increasingly hinder redox species access and impose transport limitations, which in turn can penalize interfacial charge transfer and overall electrochemical sensitivity. Therefore, an optimal protein A loading must balance Fc binding site density for robust and reproducible antibody immobilization against the transport and kinetic penalties introduced by excessive surface coverage. Based on this quantitative trade-off, 0.5 mg mL^−1^ protein A was selected for downstream biosensing, providing sufficient surface functionality for reliable oriented antibody capture while avoiding the stronger transport blocking observed at 16.7 mg mL^−1^.

**Figure 3:**
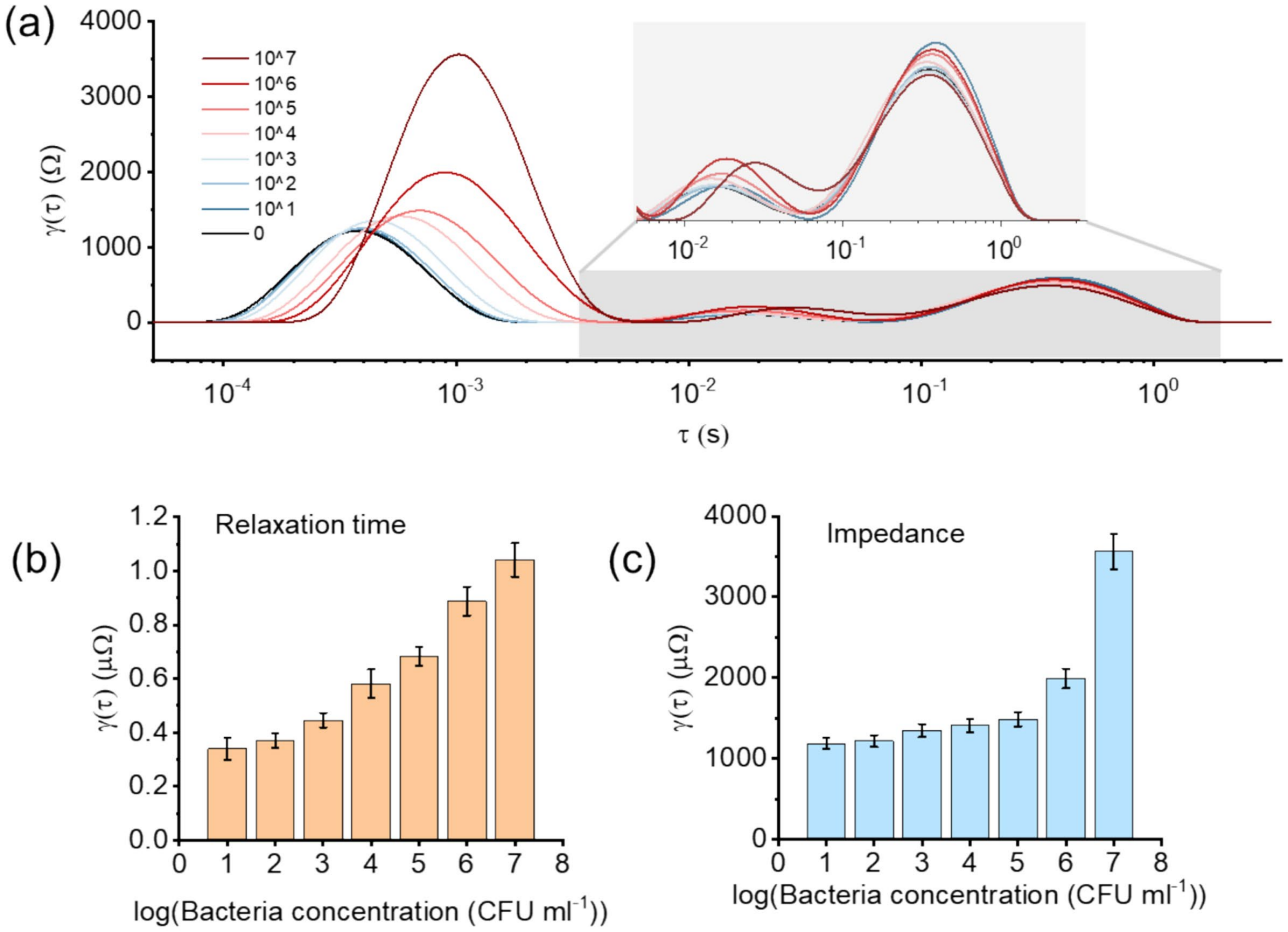
**(a)** Distribution of relaxation times (DRT) plots obtained from impedance measurements of poultry meat field samples artificially inoculated with varying concentrations of *E. coli*. Distinct peaks correspond to electrochemical processes at the electrode–electrolyte interface. **(b)** Peak-time analysis of the dominant DRT feature as a function of *E. coli* concentration, showing a clear shift in relaxation dynamics with bacterial load. **(c)** Peak-intensity analysis of the same feature, expressed as impedance (*Ω*), demonstrating concentration-dependent variations in interfacial charge transfer resistance.

To validate this biosensing sensing system, the anti-*E. coli* antibodies functionalized electrodes were incubated for 20 min with chicken breast solution with added various concentration of *E. coli O157*. Then they were tested by impedance after PBS gently flushed. The results provided clear signatures of bacterial contamination when analyzed using the DRT approach (Figure 3a). By decomposing the impedance spectra into relaxation processes, the DRT revealed multiple peaks, each representing distinct electrochemical events as discussed above. The shorter relaxation time peak (as shown in Figure 3b) related to the charge transfer process showed better correlation with bacteria concentration, suggesting that it is strongly associated with interfacial binding events between bacterial cells and the antibody functionalized electrode.

Furthermore, it showed that the relaxation time increased progressively with increasing bacterial concentration. This trend indicates that higher *E. coli* loads accelerate the interfacial charge redistribution process, likely due to increased surface coverage and enhanced dielectric polarization at the electrode surface. Such a concentration-dependent shift in relaxation dynamics highlights the sensitivity of DRT in distinguishing bacterial load levels within complex matrices such as poultry meat. However, the second peak and third peak related with double layer capacitor and diffusion process were not obvious correlated like pure protein A, which might be due to the complexity of the sample matrix.

**Figure 4:**
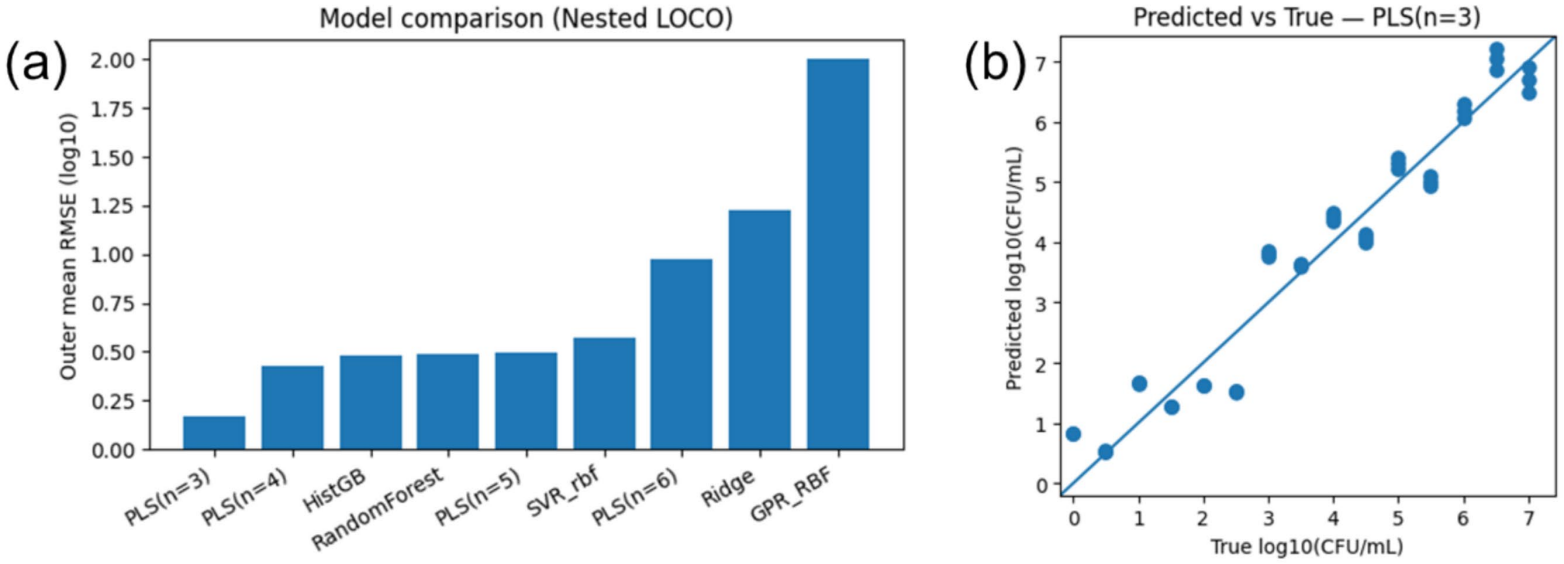
Nested LOCO cross-validation results for model comparison and prediction accuracy. **(a)** Outer-loop mean RMSE (log_10_ scale) for each model, averaged across held-out concentration levels, illustrating generalization performance. PLS(*n* = 3) achieves the lowest RMSE (~0.17), indicating the strongest prediction capability, while more complex models like GPR_RBF fail to generalize (RMSE ~2.0). **(b)** Scatter plot of true versus predicted log₁₀(CFU/mL) from the best-performing PLS (*n* = 3) model evaluated on unseen concentrations (nested LOCO), with the unity line shown for reference. The close clustering of points around the unity line indicates accurate concentration prediction across the tested range and supports robust generalization beyond the concentrations used for model training.

The DRT derived relaxation-time feature increased with bacterial concentration and enabled a conventional calibration-based analytical metric. A linear regression of relaxation time versus log₁₀(CFU/mL) yielded *τ* = 0.1395 + 0.1205·log₁₀(CFU/mL) (*R*^2^ = 0.956). Using *σ* = 0.0204 obtained from replicates at 10^1^ CFU/mL, the electrochemical LOD and LOQ (LOD = 3.3*σ*/S; LOQ = 10*σ*/S) were 0.559 and 1.693 log₁₀(CFU/mL), corresponding to approximately 3.6 × 10^1^ and 4.9 × 10^2^ CFU/mL relative to the lowest tested concentration. Notably, the relaxation-time response shows curvature across the full range (low versus high concentration regimes), indicating that a single linear calibration can introduce systematic prediction error when used for practical inference across all concentrations. Accordingly, we use ML to capture this nonlinearity for concentration prediction in application scenarios (Figure 4b).

Similarly, the impedance intensity of the dominant peak (Figure 3c) exhibited a monotonic decrease as bacterial concentration increased. This increase in resistance reflects a less efficient electron-transfer pathway in the presence of larger bacterial populations. Importantly, the combination of relaxation time and impedance intensity analyses provides a dual-parameter framework for quantifying bacterial contamination with improved robustness compared to conventional Nyquist-based interpretations.

To figure out the accurate correlation between impedance DRT spectrum and the concentration of bacteria in food samples, the machine learning models were employed and trained using these data sets (14 concentrations and each with 3 replicates, respectively). As shown in Table 1, hold-out validation has been done with high *R*^2^ (>0.95) across most models signals that features capture strong predictive structure. RMSE(log₁₀) reflects the typical log difference and fold-error estimates are especially intuitive for concentration predictions (e.g., “within 2×”). The results (35% by dataset) indicate that a Random Forest regressor performed best with *R*^2^ = 0.973 and RMSE(log₁₀) ≈ 0.351, corresponding to a ~2.3 × median fold error. SVR with an RBF kernel was a close runner-up (~2.5×). PLS Regression using four latent components achieved moderate performance (RMSE ≈ 0.382; ~2.1 × fold error) while maintaining interpretability through latent curve component analysis. Other models [e.g., Ridge, PLS(*n* = 5)] were slightly less accurate yet acceptable. Gaussian Process (RBF) failed, showing it is not suitable for this data density. The small performance gap between top models means choosing PLS vs. RandomForest becomes a balance between interpretability (PLS) and slightly higher accuracy (RandomForest). PLS(*n* = 4) is slightly behind in raw metrics but not by much (RMSE ≈ 0.382, error ≈ 2.09×), but still remains valuable, because it operates on full impedance curves using latent components, offering clear interpretability compared to tree-based models. PLS is prized in chemometrics for that reason (Table 2).

**Table 1:** Comparison of regression model performance under a 35% dataset-level hold-out validation strategy.

Model	View	*R*^2^	RMSE_log₁₀ (CFU·mL^−1^)	MAE_log₁₀ (CFU·mL^−1^)	Median fold-error
RandomForest	Tabular	0.9731	0.3514	0.3168	2.29×
SVR_rbf	Tabular	0.9709	0.3658	0.3143	2.46×
PLS(*n* = 4)	Full-curve	0.9682	0.3823	0.3446	2.09×
PLS(*n* = 5)	Full-curve	0.9630	0.4122	0.3747	2.24×
Ridge	Tabular	0.9584	0.4372	0.3952	2.37×
PLS(*n* = 6)	Full-curve	0.9525	0.4668	0.4176	2.33×
HistGB	Tabular	0.9522	0.4684	0.3514	2.50×
PLS(*n* = 3)	Full-curve	0.9510	0.4743	0.4185	3.09×
GPR_RBF	Tabular	0.0020	2.1446	1.8750	~63×

“View” indicates the representation of biosensor data used for modeling. “Full-curve” refers to training directly on the entire sensor response curve (raw time-series or spectroscopic signal), typically reduced via latent variable methods such as PLS. “Tabular” refers to models trained on engineered features extracted from the curves (e.g., peak height, slope, area), resulting in a compact feature matrix. Thus, “full-curve” leverages the entire biosensing signal, while “tabular” focuses on derived descriptors.

**Table 2:** Performance comparison of regression models using nested leave-one-concentration-out cross-validation.

Model	RMSE log₁₀ (mean)	MAE log₁₀ (mean)	Median fold-error
PLS(*n* = 3)	0.166	0.154	1.43×
PLS(*n* = 4)	0.425	0.423	2.57×
HistGB	0.481	0.469	2.93×
RandomForest	0.485	0.468	2.36×
PLS(*n* = 5)	0.494	0.482	2.68×
SVR_rbf	0.571	0.569	3.08×
PLS(*n* = 6)	0.976	0.976	9.47×
Ridge	1.223	1.221	2.70×
GPR_RBF	2.000	2.000	138.95×

Furthermore, we evaluated multiple regression models, including PLS with 3–6 latent components, SVR (RBF), Random Forest, Ridge, HistGradientBoosting (HistGB), and Gaussian Process (RBF), using a nested Leave-One-Concentration-Out (LOCO) cross-validation strategy. LOCO holds out each concentration level in turn during model evaluation, thus simulating real-world deployment with truly unseen sample levels. Nested LOCO ensures that hyperparameter tuning occurs only on training folds, preserving the integrity of performance assessment by preventing leakage of information into the testing phase. In Figure 4a the outer mean RMSE was shown, which defined as the RMSE computed on the held-out set of each outer LOCO fold and average across folds. This provides an approximately unbiased estimate of generalization to unseen concentrations. This approach is endorsed for its ability to deliver unbiased generalization estimates, particularly in chemometric sensor modeling tasks where overfitting is a prevalent concern and is consistent with standard nested-CV practice.

The results show that PLS with three latent components (PLS *n* = 3) achieved the lowest average RMSE(log₁₀) of 0.166, corresponding to a median fold-error of approximately 1.43×, outperforming all complex models. Random Forest and HistGB, despite their flexibility, exhibited RMSE values around 0.48 (fold-error ~2.4–2.9×), while Gaussian Process drastically underperformed. The predicted-vs-true plot for PLS (*n* = 3) (Figure 4b) closely follows the identity line across the dynamic range, further supporting accuracy and calibration. These findings underscore the strength of PLS’s latent-variable approach, which projects high-dimensional, noisy impedance curves into a stable low-dimensional representation, making it especially effective for generalizing across unobserved bacterial concentration levels.

The superior performance of PLS (*n* = 3) highlights the enduring value of latent-variable methods in biosensing applications. These techniques mitigate multicollinearity and noise by focusing on latent structures that covary strongest with the target, which is the principle that long emphasized in chemometrics. While ensemble learners like Random Forest and HistGB are adept at capturing non-linear trends, their modest improvement in hold-out scenarios comes at the cost of model interpretability and potential overfitting risks.

The predictivity of PLS in a stringent scenario (unseen concentration levels) speaks to its robustness and suitability for real-world biosensor deployment. Its simplicity facilitates interpretability, enabling better understanding of how impedance features relate to concentration, a considerable advantage for designing next-generation biosensing platforms for more complicated and challenged scenarios. In contrast, Gaussian Processes failed here, likely due to overfitting and limited data, indicating they may not be appropriate for high-variance datasets typical in experimental biosensing. Overall, PLS offers a balanced solution with competitive accuracy, strong generalization, and transparent modeling, which qualities paramount for advancing biosensor reliability and trust in practical settings.

**Figure 5:**
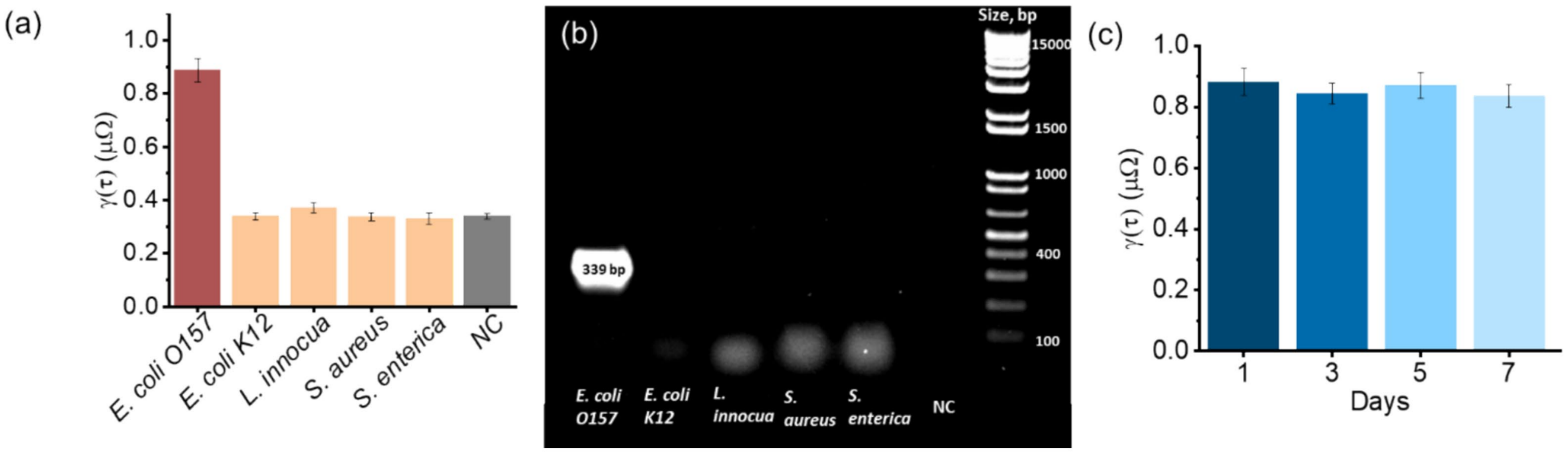
Selectivity and shelf-life evaluation of the developed biosensor. **(a)** Selectivity test against *E. coli O157:H7* and other non-target bacteria (*E. coli K12*, *Listeria innocua*, *S. aureus*, and *S. enterica*) at a concentration of 10^6^ CFU/mL, showing a significantly higher signal response only to *E. coli O157:H7*. **(b)** PCR validation confirming the presence of the 339 bp *E. coli O157:H7* amplicon and the absence of cross-reactivity with non-target strains. **(c)** Shelf-life test performed over a 7 day storage period, demonstrating stable sensor performance with negligible signal degradation.

Selectivity is essential for reliable pathogen detection in complex food matrices. As shown in Figure 5a, the biosensor produced a pronounced impedance change in response to *E. coli O157:H7* at 10^6^ CFU/mL, whereas responses to the non-target strains tested (*E. coli* K12, *Listeria innocua*, *S. aureus*, and *S. enterica*) remained close to the negative control baseline (mean ± SD, *n* = 3). This selectivity is consistent with antibody mediated recognition of bacterial surface antigens and is supported by the protein A based immobilization strategy, which promotes oriented antibody attachment and improves effective antigen binding at the electrode interface. PCR was included as an orthogonal reference to confirm the ground truth presence or absence of *E. coli O157:H7* in the prepared samples, rather than to validate the electrochemical sensing mechanism. As shown in Figure 5b, the expected 339 bp amplicon was observed only for the *E. coli O157:H7* positive sample, while no bands were detected for the non-target species, confirming correct sample labeling (target present versus absent) for the selectivity experiment. Together, these results demonstrate that the biosensor differentiates the target pathogen from closely related or co-existing bacteria in a poultry relevant context, which is critical for practical food safety screening.

Shelf-life stability is another important parameter that influences the practicality of point-of-care biosensors. As illustrated in Figure 5c, the sensor retained a consistent signal over a 7-day period, with negligible decline in detection performance. This stability is likely a result of the robust immobilization of protein A and antibodies on the gold electrode, which reduces desorption and maintains bioactivity over time. The stable baseline further ensures that the biosensor can be prepared and stored in advance, enabling its deployment for on-site testing in food production or distribution chains. Collectively, these findings confirm that the proposed biosensor not only achieves high specificity toward *E. coli O157:H7* but also maintains sufficient stability for practical field applications.

## 4 Conclusion

This study demonstrated the development of a portable electrochemical biosensor system for the rapid detection of *E. coli O157:H7* in food samples, coupled with advanced machine learning-based data analysis. The biosensor, functionalized with protein A for enhanced antibody orientation, exhibited strong electrochemical responses that were systematically evaluated using impedance spectroscopy, distribution of relaxation time (DRT), and cyclic voltammetry. To extract robust predictive performance, multiple regression and machine learning models, including PLS, Random Forest, Histogram-based Gradient Boosting, Support Vector Regression, Ridge, and Gaussian Process Regression, were applied to both leave-one-concentration-out cross-validation (LOCO-CV) and independent hold-out validation.

The ML analysis revealed that nonlinear models such as Random Forest, HistGB, and SVR offered high predictive accuracy, while linear PLS models with optimized latent variables performed consistently across datasets. Importantly, the agreement between LOCO-CV and hold-out results highlighted the generalizability of the trained models. Combined with the biosensor platform’s demonstrated selectivity, stability, and applicability to poultry field samples, these results underscore the feasibility of integrating portable biosensors with ML-driven analytics for on-site pathogen detection. This dual framework provides not only a rapid sensing tool but also a predictive modeling approach that can enhance decision-making in food safety monitoring.

Despite the rapid and selective detection demonstrated here, several limitations remain. The current workflow still requires basic sample preparation and a fixed incubation step for poultry matrices, and validation was performed on artificially inoculated samples rather than naturally contaminated field samples. In addition, broader testing across more strains/matrices and operators, and fully integrated on-device (or smartphone) DRT and ML analysis, are needed to further improve robustness and usability for point-of-need deployment.

## Data Availability

The original contributions presented in the study are included in the article/Supplementary material, further inquiries can be directed to the corresponding author/s.
