# Supplementary material for: Portable electrochemical impedance biosensing with DRT-enabled machine learning for detecting E. coli O157:H7 in poultry meat
Source: Front Artif Intell. 2026 Mar 26;9:1741144. doi: 10.3389/frai.2026.1741144 (PMC13064606; doi:10.3389/frai.2026.1741144)
Supplement: Supplementary file 1 [file Data_Sheet_1.docx]

**Machine Learning Enabled DRT-Based Immunosensor for Ultra-Sensitive Detection of *Escherichia coli O157:H7* in Poultry Samples**

Yang Tian^1,*^, Ziyu Liu^2^, Chaitanya Pallerla^3^, Siavash Mahmoudi^1^, Ramesh Bahadur Bist^1,4^, Yiting Xiao^1^, Terry Howell^1^, Jeyam Subbiah^3^, and Dongyi Wang^1,3,*^

*^1^ Department of Biological and Agriculture Engineering, University of Arkansas, Fayetteville, AR, 72701*

*^2^ Department of Animal Science, University of Arkansas, Fayetteville, AR, 72701.*

*^3^ Department of Food Science, University of Arkansas, Fayetteville, AR, 72701*

*^4^Department of Biological and Agricultural Engineering, North Carolina State University, Raleigh, NC 27695, USA*

*^*^ Correspondence: yangtian@uark.edu; dongyiw@uark.edu*

**Table S1.** Final Machine-Learning Models and Hyperparameters

| **Model** | **Input representation** | **Final hyperparameters (used in results)** | **“Number used” details** |
| --- | --- | --- | --- |
| **PLS Regression** | Full EIS/DRT curve (standardized) → latent scores | **n_components = 4** (from tested n=3–6) | 4 latent components |
| **Ridge Regression** | Engineered EIS/DRT features (standardized) | **α = 0.10**, solver = auto | 1 model (no ensembling) |
| **SVR (RBF)** | Engineered EIS/DRT features (standardized) | **C = 10.0, γ = “scale”, ε = 0.05** | 1 model |
| **Random Forest** | Engineered EIS/DRT features | **n_estimators = 400**, max_depth = None, min_samples_leaf = 1, max_features = “sqrt”, random_state = 42 | 400 trees |
| **HistGradientBoosting** | Engineered EIS/DRT features | **max_leaf_nodes = 31**, learning_rate = 0.10, max_depth = None, l2_regularization = 0.0, early_stopping = “auto”, max_bins = 255 | 1 model |
| **Gaussian Process Regression (RBF)** | Engineered EIS/DRT features (standardized) | **Kernel = σ²·RBF(ℓ)** with length_scale = 1.0 (optimized by optimizer="fmin_l_bfgs_b"), α (nugget) = 1e−6, normalize_y=True | 1 model |

**Splits and counts used for all models.**

**i) Train/test split:** 65/35 with random_state=42.

**ii) Cross-validation:** **GroupKFold (5 folds)** on the training set; the same grouping index was used across models to prevent leakage from correlated spectra.

**iii) Pipelines:** Standardization (and PLS where applicable) were fit **inside** each CV fold.

**iv) Primary metric:** RMSE on log₁₀(CFU·mL⁻¹) (regression); Accuracy/AUROC for classification variants.

**v) Sensitivity check:** leave-one-concentration-out (LOCO) curve reported separately.

**Table S2**. Approximate cost components (pilot-scale estimates)

| **Cost element** | **What it includes** | **Estimated cost range** |
| --- | --- | --- |
| Reader hardware  (reusable) | AD5941 front-end, MCU, PCB, battery/power, enclosure | ~$65 |
| Fluidic channel | Basic fluidic reactor | ~$1 |
| Disposable electrodes | Working, counter, reference electrodes plate | As low as ~1-10 cents at large scale production |
| Reagents (per test) | protein A, antibody, blockers, redox electrolyte (microliter volumes) | <$1 for each |


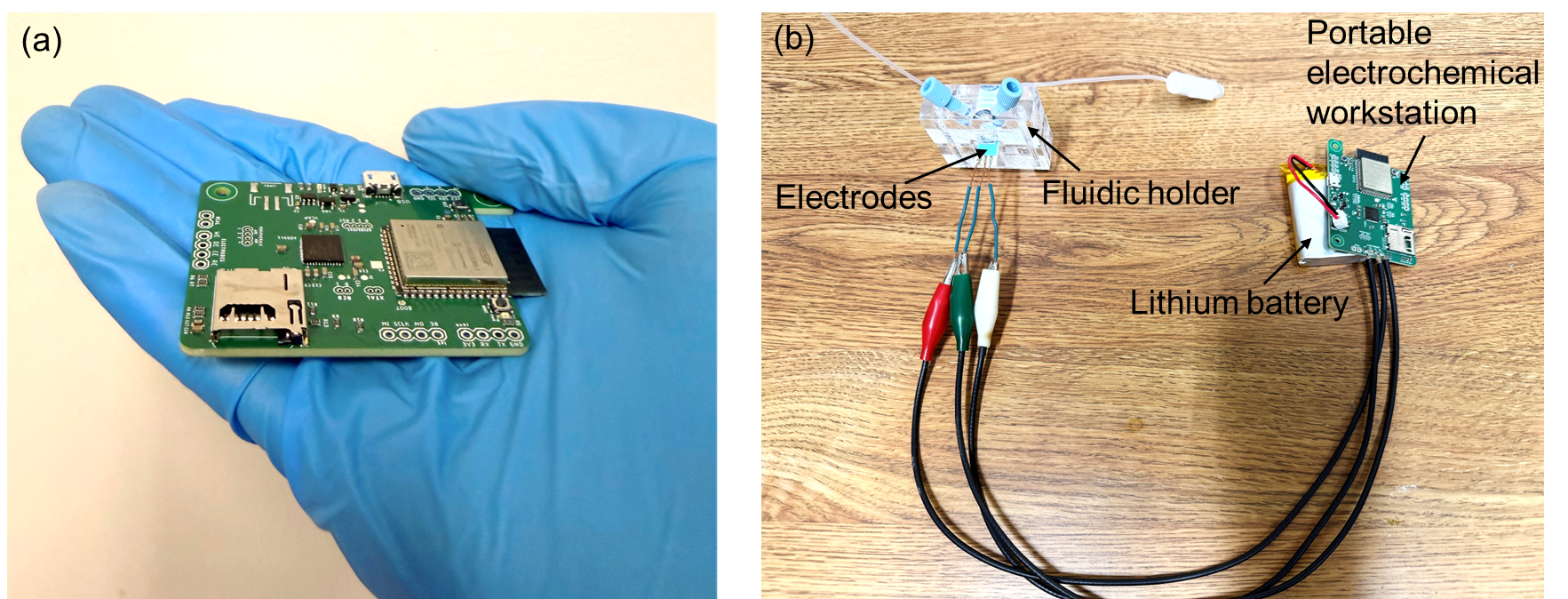


**Figure S1**. Portable electrochemical sensing platform. (a) Photograph of the custom, handheld electrochemical workstation (AD5941-based impedance front end integrated with an ESP32-S3 wireless microcontroller). (b) Fully assembled measurement setup showing the fluidic holder housing the electrode, electrical connections to the portable workstation, and battery-powered operation for EIS measurements.
